# Survival benefit of local consolidative therapy for patients with single-organ metastatic pancreatic cancer: a propensity score-matched cross-sectional study based on 17 registries

**DOI:** 10.3389/fendo.2023.1225979

**Published:** 2023-11-02

**Authors:** Xiaolong Hu, Dan Hu, Bowen Fu, Hongqi Li, Gang Ren, Hefei Liu, Jiazhao Song, Xiaoli Kang, Xuan Wang, Haifeng Pang, Chen Liu, Jianchun Zhang, Yingjie Wang

**Affiliations:** ^1^ Department of Radiation Oncology, Beijing Geriatric Hospital, Beijing, China; ^2^ Outpatient Department of Feng Tai District No.4 Retired Cadres Retreat Center, Army PLA, Beijing, China; ^3^ Department of Radiation Oncology, Air Force Medical Center PLA, Beijing, China; ^4^ Department of Radiation Oncology, Peking University Shou Gang Hospital, Beijing, China; ^5^ Center for Ion Medicine, The First Affiliated Hospital, University of Science and Technology of China, Hefei, China

**Keywords:** oligometastatic, local consolidative therapy, single-organ metastatic pancreatic cancer, radiotherapy, surgery

## Abstract

**Background:**

The continuous exploration of oligometastatic disease has led to the remarkable achievements of local consolidative therapy (LCT) and favorable outcomes for this disease. Thus, this study investigated the potential benefits of LCT in patients with single-organ metastatic pancreatic ductal adenocarcinoma (PDAC).

**Methods:**

Patients with single-organ metastatic PDAC diagnosed between 2010 - 2019 were identified from the Surveillance, Epidemiology, and End Results (SEER) database. Propensity score matching (PSM) was performed to minimize selection bias. Factors affecting survival were assessed by Cox regression analysis and Kaplan-Meier estimates.

**Results:**

A total of 12900 patients were identified from the database, including 635 patients who received chemotherapy combined with LCT with a 1:1 PSM with patients who received only chemotherapy. Patients with single-organ metastatic PDAC who received chemotherapy in combination with LCT demonstrated extended median overall survival (OS) by approximately 57%, more than those who underwent chemotherapy alone (11 vs. 7 months, p < 0.001). Furthermore, the multivariate Cox regression analysis revealed that patients that received LCT, younger age (< 65 years), smaller tumor size (< 50 mm), and lung metastasis (reference: liver) were favorable prognostic factors for patients with single-organ metastatic PDAC.

**Conclusion:**

The OS of patients with single-organ metastatic pancreatic cancer who received LCT may be prolonged compared to those who received only chemotherapy. Nevertheless, additional prospective randomized clinical trials are required to support these findings.

## 1 Introduction

Pancreatic ductal adenocarcinoma (PDAC) is identified globally as a tumor with a poor prognosis (1). Patients often present with distant metastasis during diagnosis due to the insidious symptoms of pancreatic cancer, rendering surgery a non-viable treatment option (2, 3). Consequently, systemic chemotherapy remains the mainstay therapeutic modality for metastatic PDAC. Despite the tremendous advancements of these treatments, the prognosis remains extremely poor, with a 5-year overall survival (OS) rate of only 3% (4).

In 1995, Hellman and Weichselbaum proposed the concept of oligometastatic disease as a transitional state between localized and diffused metastatic burden characterized by limited metastatic tumors in particular sites (5). Clinical studies have shown that systemic treatment combined with local consolidative therapy (LCT) to all lesions significantly improved the prognosis of oligometastatic cancer patients (6–10). Traditionally, clinical practice guidelines deem surgery a contraindication for metastatic PDAC (11, 12). However, recent studies discovered that patients with metastatic pancreatic cancer who received systemic treatment and surgery exhibited significantly improved survival compared to chemotherapy alone (13, 14). Meanwhile, a retrospective cohort study of oligometastatic pancreatic cancer (OPanc) highlighted the benefits of stereotactic ablative radiation therapy (SABR) to all active metastatic sites with a median OS of 42 months, which was greater than chemotherapy alone (15).

Despite the proven benefits of LCT in metastatic PDAC, the previous studies were performed on a small sample size. Thus, recent studies have utilized data from the Surveillance, Epidemiology, and End Results database (SEER) and reported that LCT was associated with improved OS for metastatic PDAC (16–18). Currently, surgery and radiotherapy are viable LCT options for patients with cancer, but most studies focused primarily on the contribution of surgery in OPanc and neglected the role of radiotherapy. Most surgeries are performed on selected patients with OPanc and do not fully reflect the role of LCT in general cases. Earlier studies have not addressed the bias caused by data discrimination in the SEER database. Furthermore, these studies considered multi-organ metastasis of PDAC, which may have introduced confounders into the analysis. Therefore, the purpose of this study was to analyze results from the SEER database to assess the prognostic value of the LCT to patients with single-organ metastatic PDAC who received chemotherapy.

## 2 Patients and methods

### 2.1 Data source

The data used in this study were acquired from the National Cancer Institute’s Surveillance, Epidemiology, and End Results (SEER) 17 registries Plus database (2000 - 2019) using SEER*Stat software version 8.4.0.1. The SEER data is publicly available and de-identified; thus, the data analysis does not require approval from the Institutional Ethics Committee. The researchers obtained authorization to access the database (Username: 10232-Nov2021).

### 2.2 Population study cohort

Patients with pancreatic cancer were first identified using the SEER primary site code based on the International Classification of Diseases for Oncology (ICD-0-3: C25.0 - C25.4, C25.7 - C25.9). In addition, patients with histology/behavior code 8140/3 (adenocarcinoma) or 8500/3 (invasive ductal adenocarcinoma) were selected for this study. As the database offers “SEER Combined Mets at DX liver/lung/bone/brain” information starting from 2010, this study only included patients diagnosed from 2010 to 2019. Furthermore, it should be noted that SEER has records of metastatic sites but not the number of metastatic sites.

The following patients were excluded from the study cohort: 1) patients who did not have pathological confirmation, 2) patients with pancreatic cancer that were not the first primary tumor or combined with other malignancies, 3) patients whose M stage was M0 or unclear, 4) patients with unknown T/N stage, 5) patients with multiple organ metastases (≥ 2) or metastases to organs other than lung, liver, brain, or bone, and 6) patients with single-organ metastatic pancreatic cancer who had not undergone chemotherapy. Despite the different TNM staging criteria used by the SEER database in 2010 - 2015, 2016 - 2017, and 2018-2019, the data collection for this study was not affected as the focus is on patients with metastatic pancreatic cancer. The original T/N staging criteria were used in this study to include more patients (T staging: T1/T2/T3/T4, N staging: N- and N+).

Generally, LCT is defined as receiving surgery, local radiotherapy, or surgery combined with local radiotherapy. External beam RT was the modality to receive radiation therapy; the patients whose “Radiation recode” code was “Beam radiation” were considered to receive local radiotherapy. Meanwhile, patients with Code 0 (surgery performed) were considered to have undergone surgery. Finally, the patients in this study were grouped as follows: 1) Chemotherapy group: patients who received chemotherapy; 2) Chemotherapy and LCT group: patients who received chemotherapy and LCT; 3) LCT group: patients who received LCT alone, and 4) Palliative group: patients who did not undergo chemotherapy and LCT.

### 2.3 Data collection

The following information was collected from the patient’s records: (i) Sex (Female/male), (ii) Age group (< 65 years/≥ 65 years), (iii) Race (White/black/other-unknown), (iv) Marital status (Married/divorced-separated/single/widowed/unknown-others), (v) T-stage (T1/T2/T3/T4), (vi) N-stage (N-/N+), (vii) Tumor location (Pancreatic head/pancreatic body - tail/other), (viii) Tumor size (< 50 mm/50 – 100 mm/unknown), (ix) Grade (I/II/III/IV/Blank(s)-unknown), (x) Metastasis location (Liver/lung/bone/brain), (xi) LCT (No/Yes).

### 2.4 Statistical analysis

Data analyses were performed using R v.4.2.2 statistical software (http://www.R-project.org, The R Foundation, Vienna, Austria), IBM Statistical Package for the Social Sciences (SPSS) software, version 26.0 (SPSS Inc., Chicago, IL, USA), and GraphPad Prism 9 version 9.40 (GraphPad Software, Inc., La Jolla, California USA). The categorical variables were expressed as proportions, and the differences were analyzed using Chi-square or Fisher’s exact test. The Cox regression, Kaplan-Meier plots, and the R survival package were also used for the survival analysis. A Cox proportional hazard model was applied to calculate each variable’s hazard ratio (HR) and 95% confidence interval (CI).

The multivariate Cox proportional hazard model utilized variables with p-values of < 0.2 in the univariate analysis. The multivariate analysis used backward stepwise Cox proportional hazards regression to determine the predictive factors. Survival curves were plotted via the Kaplan-Meier method and compared using the log-rank test. All the statistical tests used in this study were two-sided, and differences were considered significant at p < 0.05.

The PSM was used to adjust measured confounders, thus creating more comparable groups (19). A logistic regression analysis was undertaken using sex, age group, race, marital status, T stage, N stage, tumor location, tumor size, metastasis location, and LCT to calculate propensity scores for propensity score-matched analysis. Data relating to grade was mostly missing (blank(s)-unknown accounted for nearly 70% or more of each group), hence, unmatched in the PSM to ensure the accuracy of the study findings. The Chemotherapy group was matched to the Chemotherapy and LCT group using a 1:1 ratio with the caliper width set to 0.05 (20). The intergroup differences in categorical variables were compared using the Chi-squared test before and after matching.

## 3 Results

### 3.1 Baseline patient characteristics before PSM

**Figure 1:**
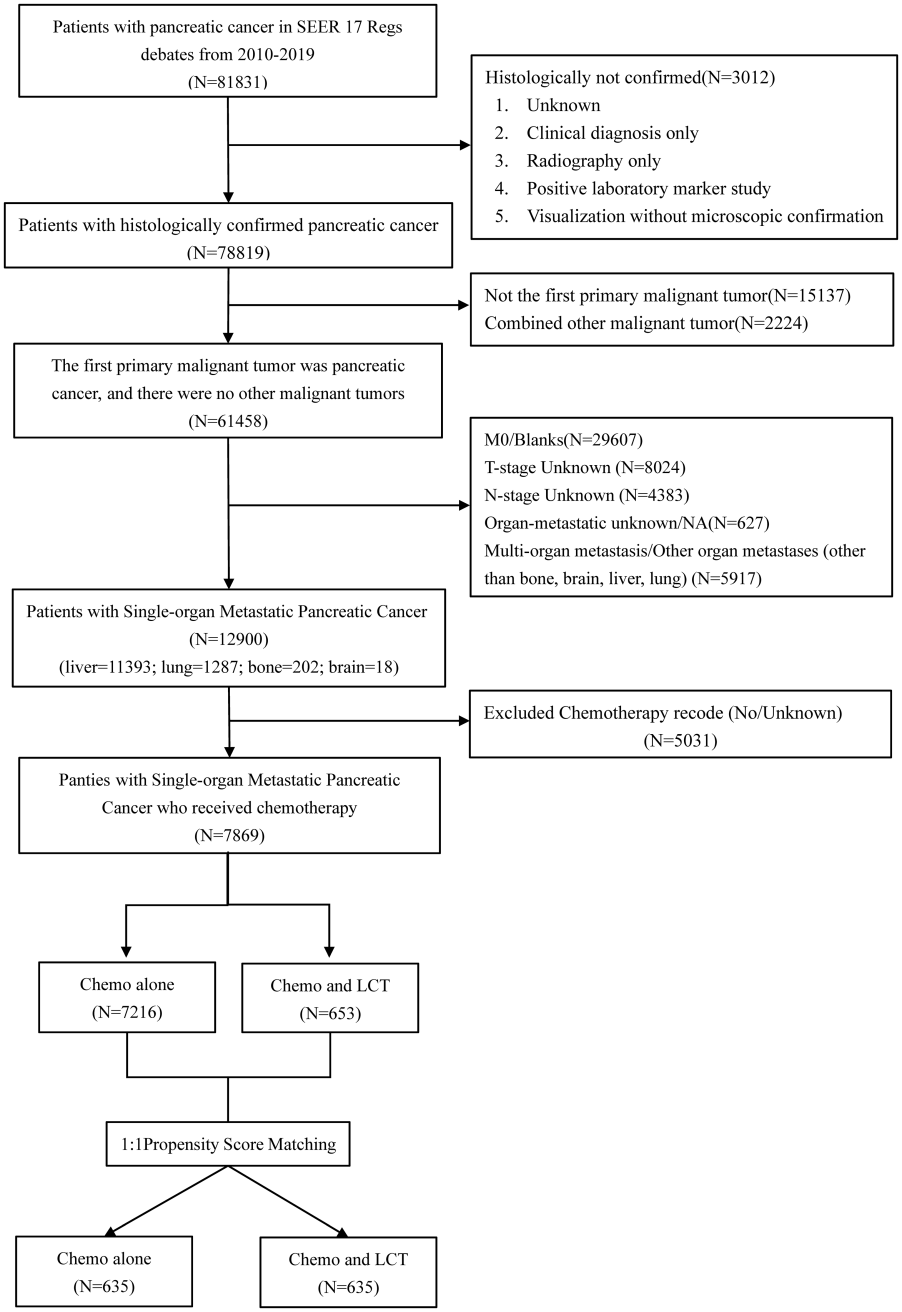
Flow chart depicting the patient selection process. SEER, Surveillance, Epidemiology, and End Results, LCT, local consolidative therapy, Chemo, chemotherapy.

A total of 81831 patients with pancreatic cancer were recorded in the SEER database from 2010 to 2019. In the present study, 12900 patients with single-organ metastatic pancreatic cancer fulfilled the inclusion and exclusion criteria (see **Table 1**; **Figure 1**). Patients with a single metastatic site often involved the liver (88.3%), followed by the lung (10.0%), followed by the bone (1.6%), and the brain (0.1%). Remarkably, N0 (n = 8634, 66.9%) was most common among patients with single-organ metastatic pancreatic cancer at the time of diagnosis. Furthermore, 7869 (61%) patients received systemic chemotherapy, and 867(6.7%) patients were treated with LCT.

**Table 1:** Characteristics of patients with single-organ metastatic pancreatic cancer.

Characteristics	Overall (N=12900)	No. of Patients(%)
Sex		
Female	5939	46.0%
Male	6961	54.0%
Age group		
< 65 years	5310	41.2%
≥ 65 years	7590	58.8%
Race		
White	10103	78.3%
Black	170 1	13.2%
Other/unknown	1096	8.5%
Marital status		
Married	7357	57.0%
Divorced/separated	1424	11.0%
Single	1956	15.2%
Widowed	1650	12.8%
Unknown/others	513	4.0%
T stage		
T1	505	3.9%
T2	4958	38.4%
T3	4602	35.7%
T4	2835	22.0%
N stage		
N^-^	8634	66.9%
N^+^	4266	33.1%
Tumor location		
Pancreatic head	5624	43.6%
Pancreatic body/tail	4871	37.8%
Other	2405	18.6%
Tumor size		
< 50 mm	9026	70.0%
≥ 50 mm	3191	24.7%
Unknown	683	5.3%
Grade		
I	137	1.1%
II	942	7.3%
III	1230	9.5%
IV	45	0.3%
Blank(s)/unknown	10546	81.8%
Liver metastasis		
No	1507	11.7%
Yes	11393	88.3%
Lung metastasis		
No	11613	90.0%
Yes	1287	10.0%
Bone metastasis		
No	12698	98.4%
Yes	202	1.6%
Brain metastasis		
No	12882	99.9%
Yes	18	0.1%
Chemotherapy		
No/unknown	5031	39.0%
Yes	7869	61.0%
LCT		
No	12033	93.3%
Yes	867	6.7%

LCT, Local Consolidative Therapy.

In the chemotherapy group, 7216 patients underwent chemotherapy alone. Meanwhile, 653 patients received chemotherapy combined with LCT in the chemotherapy and LCT group, where 246 patients underwent surgery as their primary treatment, 376 patients received radiotherapy alone, and 31 were subjected to surgery and radiotherapy. There were significant differences between the two groups before matching in terms of age, race, T-stage, N-stage, tumor location, and metastatic sites (see **Table 2**).

**Table 2:** Characteristics of patients in the chemotherapy alone and chemotherapy + LCT groups before and after the propensity score matching.

Factor	Pre-PSM			Post-PSM		
	Chemotherapy (n = 7216)	Chemotherapy+LCT (n = 653)	*p*	Chemotherapy (n = 635)	Chemotherapy+LCT (n = 635)	*p*
Sex	No. (%)	No. (%)	0.717	No. (%)	No. (%)	0.283
Female	3267 (45.3%)	301 (46.1%)		272 (42.8%)	292 (46.0%)	
Male	3949 (54.7%)	352 (53.9%)		363 (57.2%)	343 (54.0%)	
Age group			**0.006**			0.995
< 65 years	3414 (47.3%)	346 (53.0%)		335 (52.8%)	334 (52.6%)	
≥65 years	3802 (52.7%)	307 (47.0%)		300 (47.2%)	301 (47.4%)	
Race			**0.012**			0.512
White	5754 (79.7%)	521 (79.8%)		522 (82.2%)	507 (79.8%)	
Black	892 (12.4%)	63 (9.6%)		57 (9.0%)	67 (10.6%)	
Other/unknown	570 (7.9%)	69 (10.6%)		56 (8.8%)	65 (10.4%)	
Marital status			0.187			
Married	4490 (62.2%)	437 (66.9%)		434 (68.3%)	421 (66.3%)	0.726
Divorced/separated	780 (10.8%)	58 (8.9%)		50 (7.9%)	57 (9.0%)	
Single	992 (13.7%)	82 (12.6%)		87 (13.7%)	81 (12.8%)	
Widowed	679 (9.4%)	56 (8.6%)		49 (7.7%)	56 (8.8%)	
Unknown/others	275 (3.8%)	20 (3.1%)		15 (2.4%)	20 (3.1%)	
T stage			**< 0.001**			0.955
T1	254 (3.5%)	12 (1.8%)		13 (2.0%)	12 (1.9%)	
T2	2773 (38.4%)	167 (25.6%)		171 (26.9%)	163 (25.7%)	
T3	2515 (34.9%)	318 (48.7%)		305 (48.0%)	310 (48.8%)	
T4	1674 (23.2%)	156 (23.9%)		146 (23.0%)	150 (23.6%)	
N stage			**< 0.001**			0.779
N^-^	4759 (66.0%)	332 (50.8%)		320 (50.4%)	326 (51.3%)	
N^+^	2457 (34.0%)	321 (49.2%)		315 (49.6%)	309 (48.7%)	
Tumor location			**< 0.001**			0.969
Pancreatic head	3004 (41.6%)	381 (58.3%)		374 (58.9%)	370 (58.3%)	
Pancreatic body/tail	2879 (39.9%)	198 (30.3%)		191 (30.1%)	195 (30.7%)	
Other	1333 (18.5%)	74 (11.3%)		70 (11.0%)	70 (11.0%)	
Tumor size			0.506			0.979
< 50 mm	5128 (71.1%)	478 (73.2%)		470 (74.0%)	467 (73.5%)	
≥50 mm	1764 (24.4%)	147 (22.5%)		140 (22.0%)	142 (22.4%)	
Unknown	324 (4.5%)	28 (4.3%)		25 (3.9%)	26 (4.1%)	
Liver metastasis			**< 0.001**			0.898
No	786 (10.9%)	151 (23.1%)		130 (20.5%)	133 (20.9%)	
Yes	6430 (89.1%)	502 (76.9%		505 (79.5%)	502 (79.1%)	
Lung metastasis			**< 0.001**			0.875
No	6502 (90.1%)	555 (85.0%)		541 (85.2%)	538 (84.7%)	
Yes	714 (9.9%)	98 (15.0%)		94 (14.8%)	97 (15.3%)	
Bone metastasis			**< 0.001**			1
No	7149 (99.1%)	603 (92.3%)		601 (94.6%)	602 (94.8%)	
Yes	67 (0.9%)	50 (7.7%)		34 (5.4%)	33 (5.2%)	
Brain metastasis			**0.019**			1
No	7211 (99.9%)	650 (99.5%)		633 (99.7%)	632 (99.5%)	
Yes	5 (0.1%)	3 (0.5%)		2 (0.3%)	3 (0.5%)	

LCT, Local consolidative therapy; PSM, Propensity Score-Matching.

Bold values indicate significant P values.

### 3.2 Baseline patient characteristics after PSM

After a 1:1 PSM analysis, 1270 patients were included in this study (635 patients/group). The statistically significant differences in baseline characteristics between groups decreased after PSM—nonetheless, the number of patients with brain metastases was negligible between the two groups. The baseline characteristics of patients after matching are shown in **Table 2**.

### 3.3 Survival outcomes before PSM

**Figure 2:**
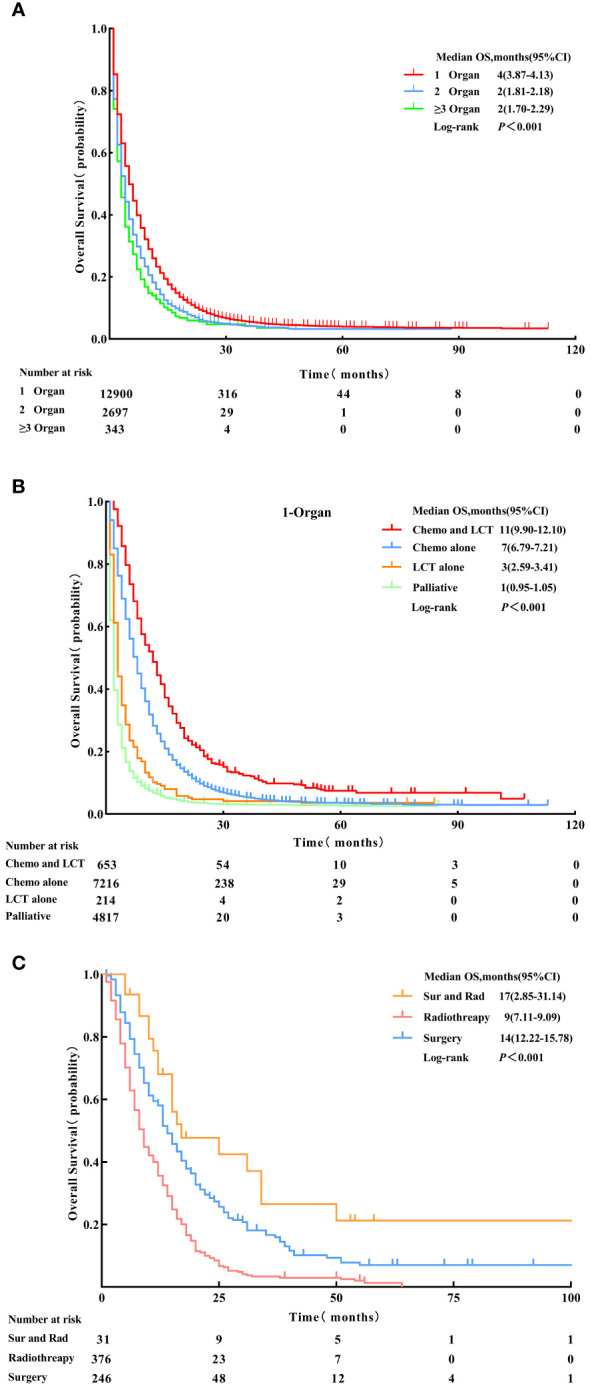
Overall survival for patients with 1-organ metastasis, 2-organs metastasis, and ≥ 3-organs metastasis. **(A)** Patients who received Chemo combined with LCT, Chemo alone, LCT alone, and palliative treatment **(B)** Patients who received Sur and Rad, Rad alone and Sur alone **(C)** before Propensity Score-Matching. LCT, local consolidative therapy, Chemo, chemotherapy, Sur, surgery, Rad, radiotherapy.

Patients with single-organ metastasis demonstrated better prognosis with a median OS of four months (95% CI: 3.87 - 4.13) than those with multiple-organ metastasis (OS, log-rank test, p < 0.001) (**Figure 2**).

The median OS for patients with single-organ metastatic pancreatic cancer in all groups were as follows: chemotherapy and LCT group = 11 months (95% CI: 9.90-12.01), chemotherapy group = 7 months (95% CI: 6.79 - 7.21), LCT group = 3 months (95% CI:2.59-3.41), and palliative group = 1 month (95% CI: 0.95 - 1.05) (see **Table 3**). The most significant improvement in survival was observed in patients who underwent chemotherapy and LCT.

**Table 3:** Median overall survival time in panties with single-organ metastatic pancreatic cancer.

Treatment group	Median survival (95% CI)	*P *(Log-rank test)
Chemotherapy and LCT	11 (9.90-12.01)	**<0.001**
Chemotherapy alone	7 (6.79-7.21)	
LCT alone	3 (2.59-3.41)	
Palliative group	1 (0.95-1.05)	

CI, Confidence Interval; LCT, Local Consolidative Therapy.

Bold values indicate significant P values.

The survival analysis using Kaplan-Meier survival curves revealed distinct differences in clinical outcomes among the four groups (OS, log-rank test, p<0.001) (see **Figure 2**). In the analysis of the chemotherapy and LCT group by local treatment modality subgroups, the median OS was 17 months (95% CI: 2.85 - 31.14) in the surgery and radiotherapy group, 14 months (95% CI: 12.22 - 15.78) in the surgery group, and 9 months (95% CI: 7.11 - 9.09) in the radiotherapy group (OS, log-rank test, p < 0.001) (see **Figure 2**).

### 3.4 Survival outcomes after PSM

**Figure 3:**
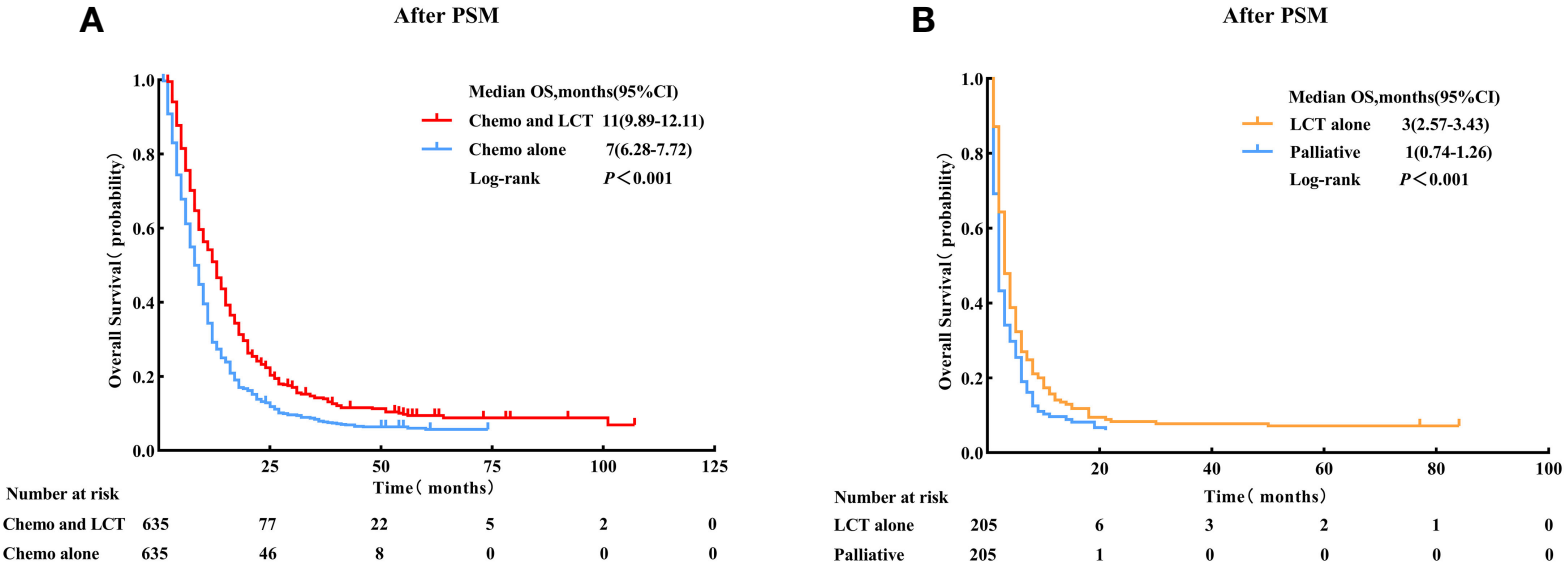
Overall survival for patients with single-organ metastatic PDAC who received Chemo combined with LCT and Chemo alone. **(A)** Patients who received LCT alone and palliative treatment, **(B)** after Propensity Score-Matching. LCT, local consolidative therapy, Chemo, chemotherapy.

The principal findings post-PSM were generally consistent with those before matching. Patients with single-organ metastatic pancreatic cancer who received chemotherapy combined with LCT had significantly improved survival than those who received chemotherapy alone (11 vs.7 months, log-rank test, p < 0.001). Meanwhile, patients who were treated with LCT alone demonstrated better survival outcomes than those who did not receive either LCT or chemotherapy (3 vs.1 month, log-rank test, p < 0.001) (see **Figure 3**).

**Figure 4:**
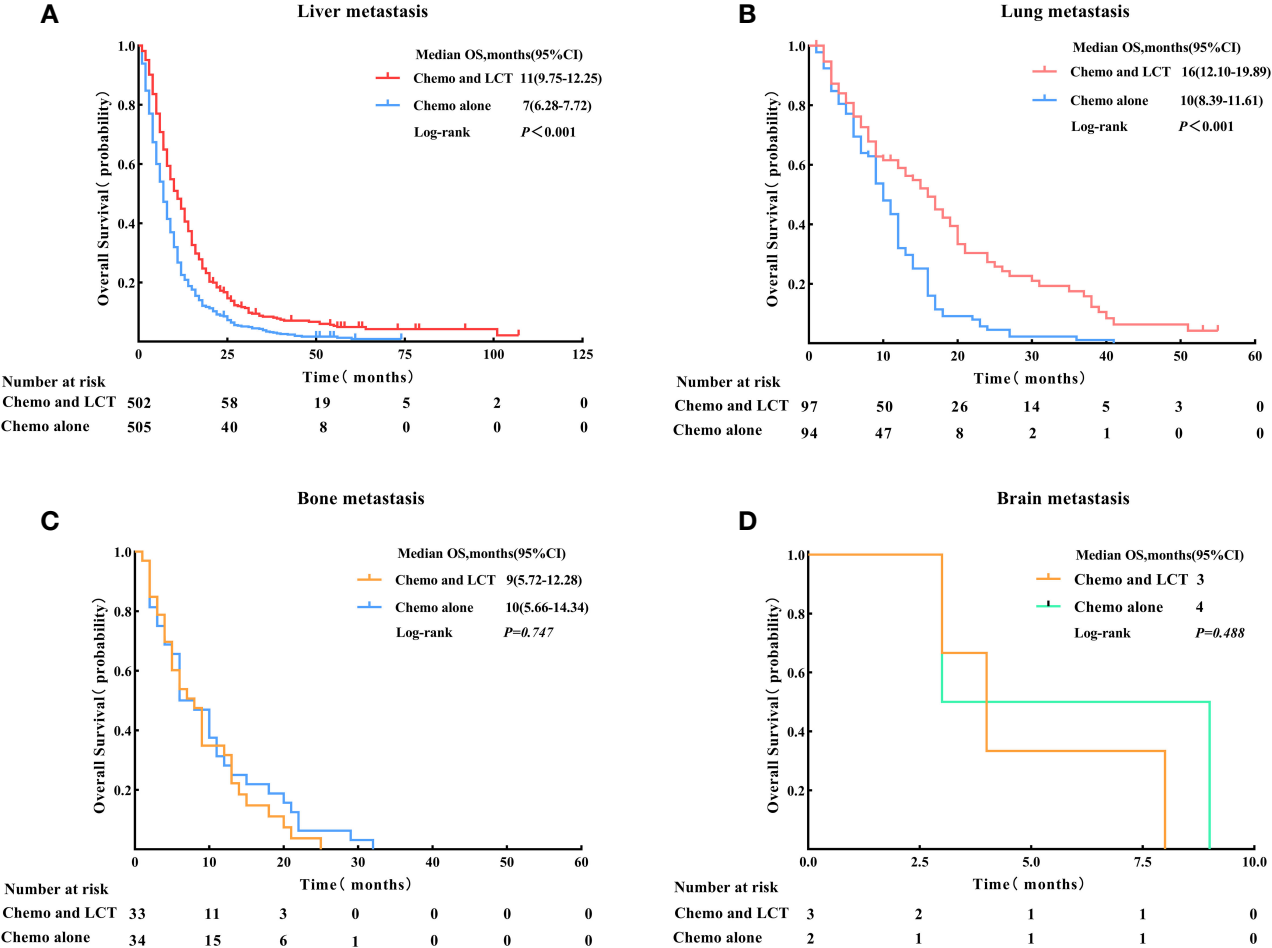
Overall survival for the type of metastatic site in patients with single-organ metastatic PDAC after Propensity Score-Matching. **(A)** Liver metastasis, **(B)** Lung metastasis, **(C)** Bone metastasis, and **(D)** Brain metastasis. LCT, local consolidative therapy, Chemo, chemotherapy.

Patients were then subdivided based on the type of metastatic site to determine which group benefited most from LCT. The results of the subgroup analysis suggested that the OS rate was significantly improved with the use of LCT for patients with liver metastasis (11 vs. 7 months, log-rank test, p < 0.001) and lung metastasis (16 vs. 10 months, log-rank test, p < 0.001) (see **Figure 4**).

### 3.5 Univariate and multivariate analyses of factors associated with OS

**Figure 5:**
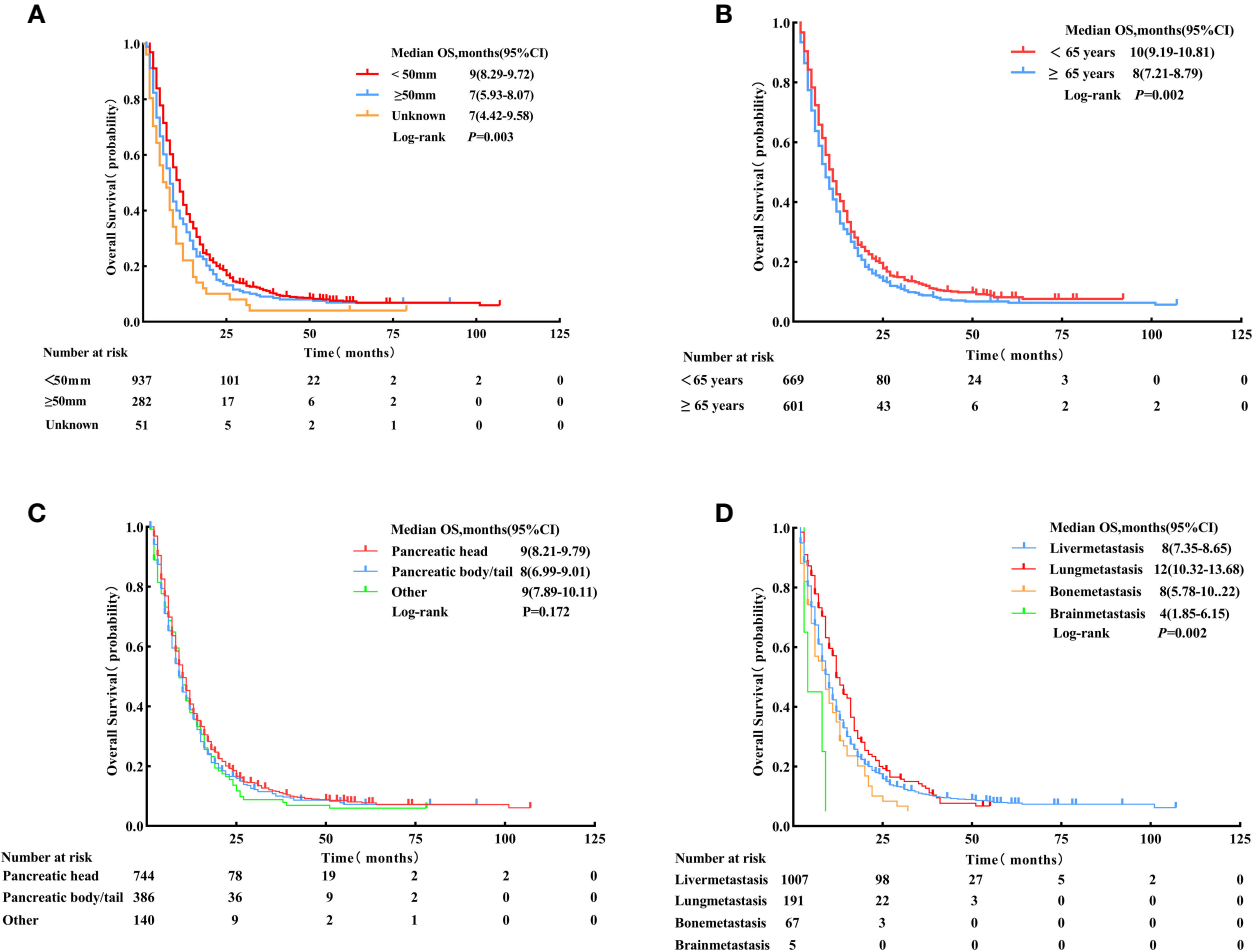
Overall survival for patients with different tumor sizes (< 50 mm, ≥ 50 mm, and unknown). **(A)** Age: < 65 years vs. ≥ 65 years, **(B)** Primary tumor location: pancreatic head, pancreatic body/tail, and other sites, **(C)** Metastasis site: liver, lung, bone, and brain, and **(D)** after Propensity Score-Matching.

The univariate Cox analysis identified the factors associated with enhanced OS, including < 65 years (HR = 0.83, 95% CI: 0.74 - 0.94, p = 0.002), smaller tumor size (< 50 mm) (HR < 50 mm vs. ≥ 50 mm = 0.80, 95% CI:0.69 - 0.92, p = 0.002), metastasis site (lung) (HR lung vs. liver metastasis = 0.81, 95% CI: 0.69 - 0.96, p = 0.015) and LCT (HR = 0.63, 95% CI: 0.55 - 0.71, p < 0.001). Meanwhile, tumor site was not a significant prognostic factor for survival (p = 0.181) (see **Table 4**; **Figure 5**).

**Table 4:** Factors associated with overall survival in univariate and multivariate analyses in panties with single-organ metastatic pancreatic cancer who received chemotherapy.

Factor	Univariable			Multivariable		
	HR	95%CI	*P*	HR	95%CI	*P*
Sex						
Female	1					
Male	0.98	0.88-1.11	0.823			
Age group						
< 65 years	0.83	0.74-0.94	**0.002**	0.83	0.73-0.94	**0.003**
≥65 years	1			1		
Race			0.442			
White	1					
Black	1.11	0.89-1.34	0.393			
Other/Unknown	0.91	0.74-1.12	0.391			
Marital status			0.113			0.097
Married	0.75	0.61-0.93	0.009	0.76	0.62-0.96	0.013
Divorced/Separated	0.86	0.65-1.15	0.316	0.89	0.66-1.18	0.372
Single	0.82	0.64-1.07	0.144	0.89	0.68-1.16	0.354
Unknown/Others	0.79	0.53-1.18	0.260	0.80	0.54-1.19	0.256
Widowed	1			1		
T stage			0.901			
T1	1					
T2	0.99	0.65-1.54	0.991			
T3	1.04	0.68-1.59	0.854			
T4	1.06	0.69-1.63	0.794			
N stage						
N^-^	1					
N^+^	1.02	0.91-1.15	0.683			
Tumor location			0.181			0.460
Pancreatic head	1			1		
Pancreatic body/tail	1.11	0.97-1.26	0.127	1.07	0.93-1.23	0.344
Other	1.15	0.95-1.39	0.166	1.12	0.91-1.37	0.286
Tumor size			**0.003**			**0.002**
<50mm	0.80	0.69-0.92	0.002	0.78	0.69-0.90	0.001
≥50mm	1			1		
Unknown	1.03	0.76-1.41	0.817	1.01	0.73-1.36	0.970
Metastases locations			**0.003**			**0.005**
Liver metastasis	1			1		
Lung metastasis	0.81	0.69-0.96	0.015	0.79	0.67-0.94	0.007
Bone metastasis	2..64	1.09-6.37	0.031	2.93	1.22-7.04	0.016
Brain metastasis	1.26	0.97-1.62	0.078	1.11	0.86-1.43	0.439
Local Consolidative Therapy						
No	1				1	
Yes	0.63	0.55-0.71	**<0.001**	0.62	0.55-0.69	**<0.001**

Multivariate analysis was performed using a backward stepwise Cox regression model.

CI, Confidence Interval.

Bold values indicate significant P values.

**Figure 6:**
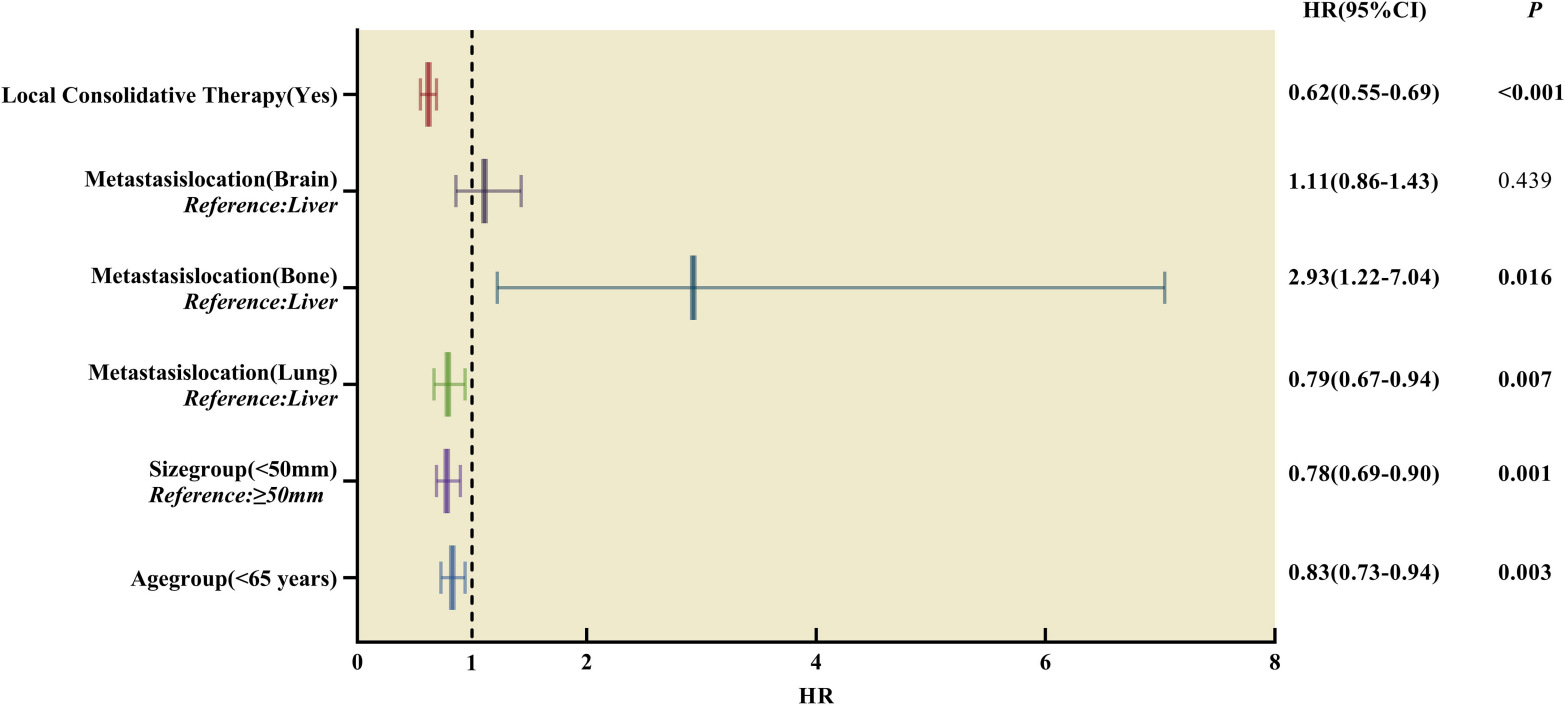
Forest plot of hazard ratios derived from multivariate Cox regression analysis of prognosis in patients with single-organ metastatic PDAC after Propensity Score-Matching. HR, hazard ratios, CI, confidence interval.

The multivariate Cox regression analysis results were consistent with the univariate analysis after matching, indicating that the parameters were independent prognostic factors for OS. The results of the multivariate analysis are presented in **Table 4**; **Figure 6**.

## 4 Discussion

Clinical and biological evidence have supported the oligometastatic state (21–23), but a consensus on the concept of oligometastatic disease has yet to be reached. Most clinical trials and the Society of Clinical Oncology agreed on the definition of 1 – 3 or 1 – 5 metastatic lesions (24). However, recent studies have shown that oligometastasis is no longer a definition but a strategy most likely to benefit from radical local therapy (25). For instance, Damanakis et al. (26) performed a retrospective analysis of 128 patients with metastatic PDAC and found that low CA19-9 levels (< 1000 U/mL), effective systemic treatment, and single-organ metastases were favorable prognostic factors for this disease. In addition, improved survival was observed in patients with single-organ metastatic PDAC than in those with multiple-organ metastasis (4 vs.2 months).

Selamir et al. (15) reported that 20 patients with synchronous or metachronous OPanc received definitive radiotherapy (median biologically effective dose 10 is 100 Gy) for all lesions and had significantly prolonged survival compared to 21 patients who did not (42 vs.18 months, p = 0.003). The current study found that definitive radiotherapy can delay systemic chemotherapy, and 17 (85%) SABR-treated patients had 6 or more months of off chemotherapy. A recent SEER data analysis of 259 patients with PDCA with liver metastases concluded that cancer-directed surgery significantly prolongs median OS by 5 - 10 months (21). These studies indicated that patients with OPanc would likely benefit from the LCT. Likewise, patients with single-organ metastatic PDAC who received chemotherapy combined with LCT in the present study had extended median OS by approximately 57% compared to those who underwent chemotherapy alone (11 vs.7 months, p < 0.001).

In this study, LCT (HR = 0.62, 95% CI:0.55 - 0.69, p < 0.001) was an independent, favorable prognostic factor for OS in patients with single-organ metastatic PDAC. Notably, 31 patients who underwent surgery in combination with radiotherapy had the best survival outcomes than those who received surgery or radiotherapy alone (17 months, p < 0.001). Therefore, it was postulated that patients with better prognoses would likely receive LCT for primary tumor and metastasis lesions, which aligned with previous reports (6–10). Moreover, surgery patients had significantly better survival outcomes than radiotherapy (14 vs. 9 months, p < 0.001). This finding may be linked to the patient selection in this study, who are generally in good condition with stable metastatic disease, high chances of recovery, lower tumor burden, and a high compliance rate (27). However, these data were unavailable in the SEER database to be analyzed in this study. Meanwhile, in the whole group of patients with single-organ metastatic PDAC, patients who received chemotherapy alone had better overall survival than those who received radiotherapy alone (7 vs.2 months, log-rank test, p < 0.001). Based on the results of this and other studies, patients with oligometastatic PDAC were more likely to benefit from radical surgery or radical radiotherapy to the primary tumor and all visible metastatic sites. To achieve the goal of a radical cure for all residual lesions, multi-modality LCT of patients with oligometastatic PDAC requires an optimal combination of radiotherapy and surgery. Moreover, patients with fewer metastatic lesions and metastatic sites more amenable to radical treatment are more likely to benefit from LCT.

Earlier studies suggested that the primary tumor site impacts the prognosis of patients with OPanc. For example, Yang et al. (28) reported that patients with liver oligometastatic pancreatic body/tail ductal adenocarcinoma who underwent resection of the primary tumor and liver metastases exhibited improved prognosis than patients who received chemotherapy alone (16.8 vs. 8 months, p = 0.003). The primary tumor site directly affects the surgical decision and the possibility of R0 resection in patients with PDAC. In this study, 153 (62.2%) patients with single-organ metastatic pancreatic cancer who underwent surgery had primary tumors located in the head of the pancreas, 78 (31.7%) in the pancreatic body/tail, and 15 (6.1%) in other sites. Meanwhile, there were no significant differences in the survival outcomes between groups (p = 0.82) and all the LCT groups (p = 0.32). Univariate and multivariate Cox regression analyses demonstrated that the primary tumor location was not a prognostic factor that may be related to highly selected patients. Earlier studies reported that tumor size was an independent prognostic factor for OS in patients with PDAC (29–31). For instance, Xu et al. (30) conducted a retrospective analysis of 221 patients with clearly resectable pancreatic cancer, and tumor size (≥ 6 cm) was a significant variable related to OS. The 7th edition of the American Joint Committee on Cancer (AJCC) Cancer Staging Manual classified tumor size by 2 cm, and the updated 8th edition classified the size of pancreatic cancer into three levels. The current study showed no differences in survival between T-stage (p = 0.48), but there was a significant difference in survival between patients with < 5 cm and ≥ 5 cm tumor sizes after the tumor reclassification (9 vs.7 months, p = 0.0017). This survival benefit may be attributed to the lower tumor burden; patients with smaller tumor sizes were more likely to receive LCT. Further analysis found that 467 (73.5%) patients with primary tumors <5 cm in the LCT group had better OS compared to 142 (24.4%) patients ≥ 5 cm (12 vs. 9 months). However, the results did not exhibit statistical significance (p = 0.31).

Multiple studies (32–34) reported that age is an important prognostic factor for patients with PDAC. For instance, Van Dongen et al. (35) conducted a population-based cohort study using data from the Netherlands Cancer Registry involving 10298 patients diagnosed with PDAC. The findings suggested that patients < 60 years mostly underwent surgery (22 vs. 14%, p < 0.001), frequently agreed to chemotherapy, and had enhanced OS (6.9 vs. 3.3 months, p < 0.001) than older patients. Meanwhile, the present study demonstrated that patients aged < 65 years were associated with better survival outcomes in univariate and multivariate analyses (HR = 0.83, 95% CI:0.73 - 0.94, p = 0.003) and improved OS compared to older patients (10 vs. 8 months, p = 0.002). This survival benefit may be attributed to the better health status of younger patients, less underlying disease, higher tolerance to chemotherapy, better immune function, and more likely to receive LCT than older patients.

In this study, only 52.6% (n = 334) of the 653 patients who received chemotherapy and LCT were younger than 65. However, further investigations were not possible due to limited data. According to the literature, the lung is an uncommon site for distant pancreatic cancer metastasis and may define a distinct biological subgroup (36). Kruger et al. (37) performed a multicenter retrospective study in PDAC with lung metastasis. They reported that the median survival time of 115 patients with PDAC and lung metastasis was 20 months, and the most favorable prognosis (28 months) occurred in 66 patients with metachronous lung metastases whom previous primary tumors underwent radical surgery. In this study, the most common site of metastatic disease was the liver (88.3%), followed by the lung (10.0%), bone (1.6%), and brain (0.1%). Patients with lung metastasis had longer median OS and better prognoses (12 months, p = 0.002) compared to other single-organ metastasis (liver, bone, and brain) in the whole group. Notably, patients with lung metastasis who received LCT had better OS compared to those who underwent chemotherapy alone (16 vs.10 months, p < 0.001). At the same time, patients with lung metastasis receiving LCT also had longer median OS (16 months, p < 0.001) compared to other single-organ metastasis (liver, bone, and brain) in the LCT group. Of these patients, 56 (57.7%) received radiotherapy, 36 (37.1%) received surgery, and 5 (5.2%) received a combination of radiation therapy and surgery.

While the SEER database provided a large patient population and long-term survival data, this study had several limitations that should be acknowledged. Firstly, the SEER database lacks complete, detailed information on patients’ general condition, tumor diagnosis, and treatment. For instance, it was challenging to discern between actual patients with oligometastatic disease due to the lack of data on the number of metastatic sites. Similarly, distinguishing synchronous from metachronous oligometastatic disease was difficult due to the lack of information on the interval between primary tumor diagnosis and the appearance of metastases. Furthermore, these diseases had different oligometastatic statuses with varying prognoses and responses to anti-cancer treatment. Third, the SEER database did not specify the radiotherapy doses and regimens or systemic treatment cycles. Nonetheless, the treatment regimens in this study were significantly homogenous as the samples covered a window from 2010 to 2019.

Chemotherapy agents and radiotherapy protocols change over time with drugs and technological advancements. Conroy et al. (38) reported that FOLFIRINOX was associated with a survival advantage (median OS) in clinical trials compared to gemcitabine (11.1 vs. 6.8 months, p < 0.001). Moreover, a phase III randomized controlled trial (39) demonstrated that nab-paclitaxel in combination with gemcitabine significantly improved the OS of patients with metastatic pancreatic cancer than gemcitabine alone (8.5 vs. 6.7 months, p < 0.001). Meanwhile, Selamir et al. (15) reported that 20 patients with OPanc who received SABR for all lesions had significantly prolonged survival compared to 21 patients who did not (42 vs. 18 months, p = 0.003). In the present study, the SEER analysis did not include the serum level CA 19-9, an important prognostic and monitoring indicator for patients with PDAC (40). An earlier study reported that a preoperative CA19-9 level of ≥ 100 U/ml significantly predicts poor prognosis after cancer surgery (41).

Information on the sequence of local therapy and systemic treatment is essential in predicting the optimal timing of LCT interventions for patients with metastatic pancreatic cancer. Based on previous randomized controlled clinical studies (7, 9, 10), systemic treatment followed by definitive local therapy for all lesions is highly beneficial for patients with oligometastatic disease. The initial systemic treatment may lead to stable or responsive disease, but the remaining tumors may contain treatment-resistant malignant cells that the maintenance systemic therapy does not eliminate. Stable disease is the first prerequisite for surgery or definitive radiotherapy to all residual tumor sites. However, suppose the patient has known presumably symptomatic brain metastases or a series of bone-related events that seriously affect the quality of life. In that case, it is necessary to consider the timing of local therapy. Additionally, the SEER database was unavailable for information relating to underlying diseases, insurance information, and smoking status. These factors can potentially influence patients’ survival and treatment decisions. Lastly, the SEER database does not include data on the treatments for recurrences or progression that could contribute to the decision-making for palliative patients and those who have undergone chemotherapy and radiation.

There is increasing promising evidence of the clinical benefits of LCT in oligometastatic disease. However, mechanism research and prospective studies on oligometastatic PDAC are sparse. The differential between non-oligometastatic PDAC and true oligometastatic PDAC is a real challenge for physicians. Identification of the true oligometastatic state plays a vital role in clinical decision-making, which is significant in determining the therapeutic option. In addition, tumor immunotherapy has revolutionized cancer treatment and has established itself as an important pillar of oncological therapy. Shi Y et al. (42) conducted a study on the ferroptosis regulator and its association with the immune microenvironment and programmed cell death ligand 1 (PD-L1) in pancreatic adenocarcinoma, and it was found that FANCD2 could be effective for prognostic recognition, immune efficacy evaluation, and mRNA vaccine for patients with pancreatic adenocarcinoma. Meanwhile, Wu L et al. (43) summarized preclinical and clinical studies regarding the synergic effect of radiotherapy combined with immune checkpoint inhibitors, especially SBRT irradiation to the tumor. Moreover, Bauml JM et al. (44) reported that after locally ablative therapy for oligometastatic non-small cell lung cancer, pembrolizumab improved PFS with no reduction in quality of life (median PFS: 19.1 months).

## 5 Conclusion

The SEER database analysis in this study suggested that patients with single-organ metastatic pancreatic cancer (liver, lung, bone, brain) who received chemotherapy combined with LCT may prolong their OS more than chemotherapy alone. Nevertheless, further studies should be conducted to support LCT and the optimal combination of systemic treatment and LCT as treatment options for this disease and identify patients who would benefit most from the treatment.

## Data availability statement

The raw data supporting the conclusions of this article will be made available by the authors, without undue reservation.

## Ethics statement

Ethical review and approval were waived for this study due to the data being publicly available and anonymous.

## Author contributions

(I) Conception and design: YW, XH. (II) Administrative support: DH, JZ. (III) Provision of study materials or patients: GR, CL, XW, BF. (IV)Collection and assembly of data: XH, HQL, HP, HFL, JS. (V) Data analysis and interpretation: XH, HQL, XK. (VI) Manuscript writing: XH, DH. All authors contributed to the article and approved the submitted version.
